# Gut-Brain Axis: Investigating the Effects of Gut Health on Cognitive Functioning in Adults

**DOI:** 10.7759/cureus.64286

**Published:** 2024-07-10

**Authors:** Muddsar Hameed, Fatima Noor, Hamza Hussain, Raja Gohar Khan, Shahbaz Khattak Haroon Ur Rashid, Spogmaye Haroon Ur Rashid, Alina Atiq, Hassan Ali, Seerat e Rida, Mahrukh Anwar Abbasi

**Affiliations:** 1 Department of Clinical Psychology, Shifa Tameer-e-Millat University, Islamabad, PAK; 2 Department of Internal Medicine, Foundation University Medical College, Islamabad, PAK; 3 Department of Internal Medicine, Shifa International Hospital, Islamabad, PAK; 4 Department of Emergency Medicine, Pakistan Ordnance Factory Hospital, Islamabad, PAK; 5 Department of Internal Medicine, Al Nafees Medical College and Hospital, Islamabad, PAK; 6 Department of Psychology, Birmingham City University, Birmingham, GBR; 7 Department of Internal Medicine, Bahria University Medical and Dental College, Karachi, PAK

**Keywords:** cognitive performance, processing speed, memory, microbiota, cognitive functioning, gut health, gut-brain axis

## Abstract

Introduction: The gut-brain axis is a bidirectional communication network linking the gastrointestinal tract and the central nervous system via neuronal, hormonal, and antibody signaling pathways. Central to this connection is gut health, encompassing the balance and functionality of gut microbiota, which significantly impacts on mental and cognitive health. This study investigates the association between gut health and cognitive functioning in adults, highlighting the mechanisms by which gut microbiota influence brain health.

Objective: To examine the effects of gut health on adult cognitive performance, with a focus on the processes by which gut microbiota impacts brain health.

Methods: A quantitative cross-sectional study was conducted in Islamabad from January 2024 to April 2024, involving 140 adult participants. Data were collected using a comprehensive 16-item gut health questionnaire and the cognition self-assessment rating scale (C-SARS). The psychometric properties of these scales were assessed, and the data were analyzed using Statistical Product and Service Solutions (SPSS, v26; IBM SPSS Statistics for Windows, Armonk, NY). Analytical and descriptive statistics, including regression, chi-square, independent sample t-tests, and mean and standard deviation, were applied.

Results: The study found moderate associations between gut health and cognitive performance, particularly in memory and processing speed (R² = 0.17, β = -1.9, p = 0.12 for general cognition; R² = 0.01, β = -0.98, p = 0.02 for memory; R² = 0.03, β = -0.18, p = 0.03 for processing speed). Gender and marital status differences were significant, with males exhibiting better gut health scores than females (M = 34.1, SD = 3.2 vs. M = 31.2, SD = 3.2, p = 0.00), and singles showing better cognitive performance compared to married individuals (M = 9.4, SD = 5.4 vs. M = 6.5, SD = 3.7, p = 0.03).

Conclusion: The study highlights significant associations between gut health and cognitive functions, suggesting that gut microbiota composition can influence cognitive performance. Gender and marital status differences underscore the need to consider individual differences in gut-brain axis research. Future studies should replicate these findings in larger samples and explore gut microbiota-targeted interventions for cognitive health enhancement.

## Introduction

The gut-brain is a bidirectional communication network that links the gastrointestinal tract and the central nervous system by using the neuronal, hormonal, and antibody signaling pathways. Central to this connection is gut health, which encompasses the balance and functionality of gut microbiota. The gut microbiota, a diverse group of bacteria that reside in the gastrointestinal tract, has been connected to effects on mental and cognitive health in addition to its role in maintaining this communication network [1]. New findings in the field of microbiome research have highlighted the substantial impact of gut bacteria on brain function. The gut microbiota influences the central nervous system by generating neurotransmitters, managing the immune system, and modifying the hypothalamic-pituitary-adrenal axis [2]. An imbalance in the gut microbiota known as dysbiosis has been linked to several neurological and psychiatric conditions, such as anxiety, depression, and cognitive decline [3,4].

Short-chain fatty acid (SCFA) generation and synthesis, including acetate, propionate, and butyrate, is one of the more significant ways that gut bacteria can alter cognitive function. It has been demonstrated that these metabolites, which are created when gut bacteria ferment food fibers, can pass across the blood-brain barrier and have neuroprotective properties. Butyrate has been shown to support cognitive health by promoting neurogenesis, enhancing synaptic plasticity, and lowering neuroinflammatory responses [5,6].

The gut microbiota is significantly impacted by the food and lifestyle choices of the developed world, which in turn influences cognitive functions [7]. Diets high in processed carbs and added sugars, poor in fiber, and consuming little in the way of probiotics might negatively impact gut flora and may be linked to cognitive problems [8]. The gut microbiota has long been nourished by fermented foods, probiotic products containing a range of beneficial strains, and strains that specifically promote optimal brain function through gut-brain axis stimulation [9]. They aid in digestion, encourage a balanced population of good bacteria in the gut, and boost their growth and activity. Taking probiotics and prebiotics (symbiotics) at the same time can help maintain a balanced population of good bacteria in the gut and boost the functions of the immune and digestive systems [10].

Research has shown that changes in gut microbiome affect cognitive performance in both healthy and sick populations and are impacted by modifications to the alteration of the gut microbiota [11,12]. Bacteria can also interact with the central nervous system by (1) producing and secreting entire neurotransmitters in the form of metabolites/fractions, such as serotonin; and (2) secreting amino acids and other compounds, such as folate and SCFAs, or combinations thereof [13,14]. However, because the gut microbiota is flexible, a variety of external variables can affect it, including drugs, healthy vs bad diet, chemicals, and stress (from high-stress jobs to poor sleep) [15].

Empirical data demonstrating the connection between gut microbiota and cognitive performance has been shown in several investigations. Jiang et al., for instance, discovered that patients with major depressive disorder had significantly different gut microbiota compositions from healthy controls, with higher pro-inflammatory and lower anti-inflammatory bacterial levels [16]. In a similar vein, Allen et al.'s randomized controlled experiment showed that giving healthy participants a multi-strain probiotic pill enhanced their cognitive function and decreased their signs of anxiety and sadness [17].

Given these results, the purpose of this study is to examine how gut bacteria affect adult cognitive performance, with a particular emphasis on the processes by which gut microbiota affects brain health. By elucidating these mechanisms, we hope to contribute to the development of microbiota-targeted therapies for cognitive enhancement and mental health improvement.

Rationale of the study

The rationale for this study stems from the increasing recognition of the gut-brain axis as a critical pathway influencing cognitive functioning and mental health. Understanding the relationship between gut bacteria and cognitive performance can provide insights into potential mechanisms underlying cognitive impairments and offer new avenues for therapeutic interventions. This study's significance lies in its potential to identify specific gut microbiota profiles associated with cognitive health, contributing to the development of microbiota-targeted therapies for cognitive enhancement and mental health improvement. By elucidating the intricate connections between gut health and cognitive functions, this research aims to advance our knowledge of the gut-brain axis and promote strategies for maintaining cognitive well-being through dietary and lifestyle modifications.

## Materials and methods

Study design and setting

We conducted a quantitative, cross-sectional study in Islamabad to investigate the association between gut health and cognitive function in the general population. Adult participants (aged 18 and above) who provided electronic informed consent were enrolled in the study. Data collection took place from January 2024 to April 2024. Ethical approval was obtained in January 2024, following which data collection commenced. Participants above the age of 18 who provided informed consent were included in the study. However, individuals with severe gut problems, psychiatric and cognitive issues, or those already using psychiatric medication were excluded from the study.

Sample size and technique

Using the WHO sample size calculator with a confidence interval (CI) of 95%, an anticipated population proportion of 0.90, and an absolute precision of 0.05, our calculated sample size was 139, which we rounded to 140. A non-probability convenience sampling technique was employed to collect data from 140 participants in Islamabad due to resource and time constraints.

Data collection tools and procedure

To assess participants' gut health and related behaviors, we developed a comprehensive questionnaire comprising 16 items (Appendix Table 6). The questionnaire includes items on dietary habits, such as the frequency of consuming fruits, vegetables, whole grains, prebiotics, nuts, seeds, and fermented foods, as well as lifestyle factors such as meal frequency, water intake, and physical activity. Specific health-related behaviors, including smoking and alcohol consumption, were also covered. Additionally, the questionnaire assesses gastrointestinal symptoms, diagnosed conditions, recent medical procedures, and an overall gut health rating on a scale of 1 to 3. The questionnaire items were designed based on existing literature on gut health and dietary assessments. Each item was crafted to capture a specific aspect of gut health, ensuring comprehensive coverage of relevant factors influencing gut microbiota composition and function. Participants were asked to respond to each item based on their typical behavior and experiences over the past month [18-20].

The full questionnaire is included in the Appendix (Table 7), and the scoring system for the questionnaire is as follows: excellent gut health practices are indicated by scores ranging from 36 to 48 points, moderate practices by scores from 24 to 35 points, and poor practices by scores from 16 to 23 points. Cronbach’s alpha for this questionnaire is 0.68. For cognitive assessment, we used the cognition self-assessment rating scale (C-SARS), developed by Dr. Henry Nasrallah. This scale evaluates various cognitive functions, including attention, memory, executive functions, processing speed, and social cognition. Cronbach's alpha for C-SARS is 0.80. The C-SARS consists of 12 items where individuals rate the frequency and impact of their cognitive difficulties. Scoring involves assigning 0 points for "Rarely," 1 point for "Sometimes," 2 points for "Often,” and 3 points for "All the time." A total score of 0 indicates normal cognition, 1-11 suggests mild cognitive problems, 12-22 indicates moderately severe cognitive deficits, and 23-33 denotes severe cognitive impairment, warranting a full cognitive assessment [21].

We developed an online Google Form to collect data, which included demographic questions, a gut health questionnaire, and a C-SARS. Participants were asked to provide their responses based on their typical behavior and experiences over the past months. An online consent form was included to ensure that participants provided informed consent before participating in the study. Ethical approval was taken from the Institutional Review Board and Ethical Committee of Brain Wave Research Center (BRC-IRB-0015) before commencing the study. Participants were informed about the aim of the study and their volunteer participation along with the freedom to refuse anytime at the start or during the study. Confidentiality, anonymity, and self-respect of all the participants were maintained. Statistical Product and Service Solutions (SPSS, v26, IBM SPSS Statistics for Windows, Armonk, NY), a statistical package for social sciences, was used to analyze the data. The data were analyzed using both analytical and descriptive statistics, including regression, chi-square, independent sample t-test, and mean and standard deviation. A significance threshold of p < 0.05 was applied.

## Results

Table 1 presents the psychometric properties of five scales, including the number of items, skewness, kurtosis, and Cronbach's alpha. The gut health questionnaire, comprising 15 items, demonstrates a skewness of -0.02, kurtosis of -0.3, and Cronbach's alpha of 0.63, indicating moderate internal consistency. The C-SARS with 12 items shows a skewness of 0.74, a kurtosis of 0.56, and a Cronbach's alpha of 0.80, reflecting good internal consistency. The memory scale, with four items, has a skewness of 0.82, a kurtosis of 0.29, and Cronbach's alpha of 0.76, also indicating good internal consistency. The executive function scale, consisting of three items, exhibits a skewness of 0.91, a kurtosis of 0.97, and Cronbach's alpha of 0.63, reflecting moderate internal consistency. Lastly, the social cognition scale, with three items, shows a skewness of 0.6, a kurtosis of 0.4, and Cronbach's alpha of 0.71, indicating acceptable internal consistency.

**Table 1:** Psychometric properties of scales C-SARS = cognition self-assessment rating scale

Scales	No of Items	Skewness	Kurtosis	Cronbach’s Alpha
Gut Health Questioner	15	-0.02	-0.3	0.63
C-SARS	12	0.74	0.56	0.80
Memory	4	0.82	0.29	0.76
Executive Function	3	0.91	0.97	0.63
Social Cognition	3	0.6	0.4	0.71

Table 2 represents an analysis of the relationships between various demographic and health-related variables across different age groups (young adults, middle adults, and old adults), gut health status (poor, moderate, and excellent), and cognitive functioning (normal cognition, mild, moderate, and severe deficits). The gender distribution indicated that males constituted 48 (34.3%) of the sample, with a significant age distribution (p = 0.04, χ2 = 1.8) predominantly among young adults. Males primarily had moderate gut health (p = 0.00, χ2 = 15.5) and were largely within the normal cognition category. Females represented 92 (65.7%) of the participants, with the majority being young adults. They also mainly reported moderate gut health and normal cognition, although no significant differences were observed in their cognitive functioning across the different age groups (p = 0.53, χ2 = 2.1). In terms of education, high school graduates comprised 66 (47%) of the sample, with a significant concentration in young adults (p = 0.00). They had moderate gut health predominantly and showed significant cognitive deficits (p = 0.01, χ2 = 9.2). Bachelor's degree holders accounted for 58 (41%), mostly young adults, with moderate gut health and normal cognition. Master's degree holders were 16 (11%), with a notable distribution in middle and old adults, all of whom had moderate gut health and normal cognition. Marital status analysis revealed that singles constituted 108 (77.1%) of the participants, significantly young adults (p = 0.00, χ2 = 9.6). Singles mainly had moderate gut health and normal cognition, though with some mild cognitive deficits. Married individuals were 32 (22.9%), mainly middle and old adults, showing moderate gut health and normal cognition, but with significant differences in cognitive deficits (p = 0.02). Employment status indicated that 108 (77.1%) of the participants were employed, predominantly young adults (p = 0.00, χ2 = 9.6). Employed participants mainly had moderate gut health and normal cognition, with some showing mild cognitive deficits. Unemployed individuals were 32 (22.9%), with a notable distribution in middle and old adults. They also had moderate gut health and normal cognition, with significant differences in cognitive functioning (p = 0.02). These findings highlight the significant associations between demographic variables, gut health, and cognitive functioning, suggesting that younger individuals, singles, and employed participants generally exhibit better gut health and cognitive function. The chi-square values indicate the strength of these associations, with notable significance in gender, education, marital status, and employment status across different age groups and health outcomes.

**Table 2:** Descriptive statistics of demographic variables f=frequency; %=percentage; p=level of significance; p-values calculated using the chi-square test; the significance level is set at p < 0.05.

Variables	f (%)	Young Adults	Middle Adults	Old Adults	p	χ^2^	Poor Gut Health	Moderate Gut Health	Excellent Gut Health	p	χ^2^	Normal Cognition	Mild Deficit in Cognition	Moderate Deficit in Cognition	Severe Deficit in Cognition	p	χ^2^
Gender																	
Male	48 (34.3)	44	2	2	0.04	1.8	0	32	16	0.00	15.5	0	35	11	2	0.53	2.1
Female	92 (65.7)	77	9	6			1	84	7			3	69	18	2		
Education																	
High school	66 (47)	63	3	0	0.00	72	1	54	11	0.28	5.0	2	51	9	4	0.01	9.2
Bachelor’s	58 (41)	55	1	2			0	46	12			1	40	17	0		
Master’s	16 (11)	3	7	3			0	16	0			0	13	3	0		
Marital status																	
Single	108 (77.1)	105	2	1	0.00	47	1	87	20	0.36	1.8	1	76	27	4	0.02	9.6
Married	32 (22.9)	16	9	7			0	29	3			2	28	2	0		
Employment status																	
Employed	108 (77.1)	105	2	1	0.00	47	1	87	20	0.03	1.8	1	77	27	4	0.02	9.6
Unemployed	32 (22.9)	16	7	7			0	29	3			2	28	2	0		

**Figure 1:**
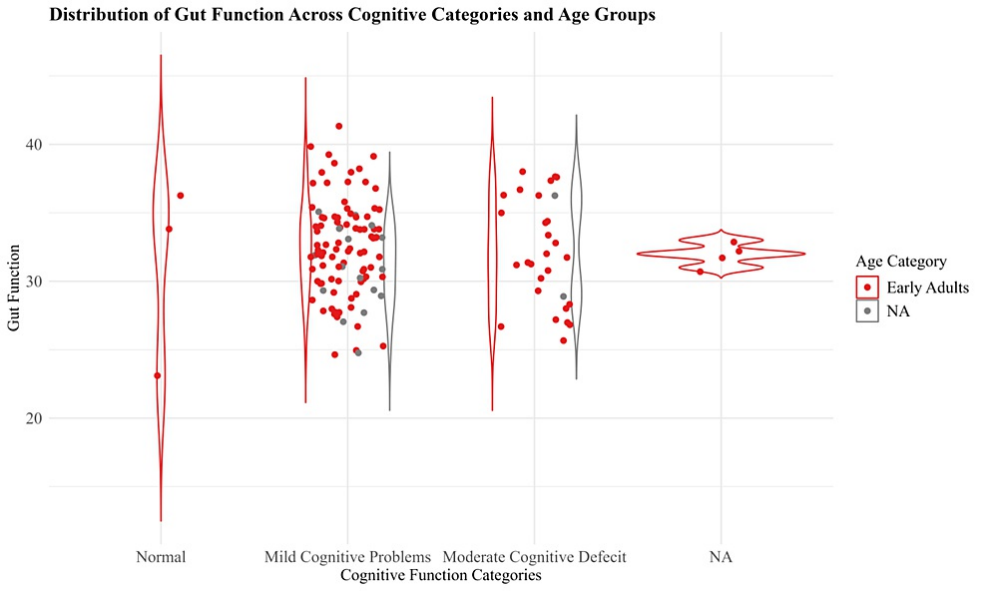
Distribution of gut function across cognitive categories in early adults

The violin plot in Figure 1 illustrates the distribution of gut function across different cognitive function categories, specifically for early adults. Gut function is plotted on the y-axis, while cognitive function categories (normal, mild cognitive problems, moderate cognitive deficit) are on the x-axis. The red color represents early adults, and the gray color represents the NA group, which includes middle and older adults who were not considered due to lower frequency. Each category shows the spread of data points, with density distributions highlighting the variability within each group.

Table 3 presents the inter-correlation coefficients among study variables. Significant findings include a strong negative correlation between cognition and memory (r = -0.83, p < 0.01), a strong positive correlation between cognition and executive function (r = 0.80, p < 0.01), and positive correlations of cognition with processing speed (r = 0.39, p < 0.01) and social cognition (r = 0.63, p < 0.01). Gut health shows small negative correlations with cognition (r = -0.13, p < 0.05) and processing speed (r = -0.18, p < 0.05). Executive function positively correlates with memory (r = 0.55, p < 0.01), processing speed (r = 0.19, p < 0.05), and social cognition (r = 0.34, p < 0.01). Additionally, processing speed and social cognition are positively correlated (r = 0.50, p < 0.01), highlighting the intricate relationships among cognitive functions and health factors.

**Table 3:** Intercorrelations between study variables *=p<0.05, **=p<0.01 considered significant; p-values calculated using Pearson correlation the significance levels are set at p < 0.05 and p < 0.01.

Variables	Cognition	Gut Health	Attention	Memory	Executive Function	Processing Speed	Social Cognition
Cognition	-	-	-	-	-	-	-
Gut health	-0.13^*^	-	-	-	-	-	-
Attention	-0.05	0.14	-	-	-	-	-
Memory	-0.83^**^	-0.09	-0.05	-	-	-	-
Executive function	0.80^**^	-0.03	-0.00	0.55^**^	-	-	-
Processing speed	0.39^**^	-0.18^*^	-0.03	0.08	0.19^*^	-	-
Social cognition	0.63**	-0.12	-0.04	0.30^**^	0.34^**^	0.50^**^	-

Table 4 represents the independent sample t-tests to compare gender and marital status across various cognitive and health-related variables. The comparison of gender across various study variables reveals that there are no significant differences between males and females in cognition, attention, executive function, memory, social cognition, and processing speed. Specifically, while males show slightly higher mean scores in cognition compared to females, the difference is not statistically significant (t = 1.4, p = 0.16). Gut health, however, shows a significant difference with males scoring higher than females, indicating better gut health among males (t = 5.0, p < 0.001), with a large effect size (Cohen’s d = 0.89). This suggests that gut health is notably different between genders, while other cognitive and social variables remain comparable. When comparing marital status, significant differences are found in cognition, executive function, memory, and attention. Single individuals score higher in cognition than married individuals, with a significant t-value (t = 2.91, p = 0.03) and a moderate effect size (Cohen’s d = 0.65). Similarly, single individuals perform better in memory compared to married individuals, with a significant difference (t = 3.47, p < 0.001) and a large effect size (Cohen’s d = 0.70). Gut health is also better among single individuals compared to married ones, with a significant difference (t = 1.01, p = 0.02) and a small effect size (Cohen’s d = 0.20). These findings suggest that marital status influences certain cognitive functions and gut health, with single individuals generally performing better in these areas. Overall, the data indicate that, while gender does not significantly impact most cognitive and social variables, gut health shows notable differences between males and females. In contrast, marital status significantly affects several cognitive functions and gut health, with single individuals showing better outcomes.

**Table 4:** Comparison of gender and marital status across study variables using independent sample t-tests M=mean, SD=standard deviation, LL=lower limit, UL=upper limit, CI=confidence interval; p-values calculated using independent sample t-tests; the significance level is set at p < 0.05.

Gender Comparison									
Variables	Male		Female		-	-	95%CI		-
-	M	SD	M	SD	t(138)	P	LL	UL	Cohen’s d
Cognition	9.6	5.4	8.3	5.0	1.4	0.16	-0.52	3.12	0.25
Gut health	34.1	3.2	31.2	3.2	5.0	0.00	1.7	4.0	0.89
Attention	2.1	0.58	2.1	0.39	-0.07	0.93	-0.17	0.15	-0.01
Executive function	2.6	1.9	2.0	1.7	1.6	0.05	-0.90	1.18	0.30
Memory	3.8	2.6	3.3	2.5	1.1	0.24	-0.37	1.42	0.21
Social cognition	1.4	1.5	1.3	1.4	0.46	0.02	-0.39	0.63	0.08
Processing speed	0.68	0.77	0.79	0.73	0.72	0.42	-0.36	0.15	-0.14
Comparison among marital status									
Variables	Single		Married		-	-	95%CI		-
-	M	SD	M	SD	t(138)	P	LL	UL	Cohen’s d
Cognition	9.4	5.4	6.5	3.7	2.91	0.03	0.95	4.9	0.65
Gut health	32.4	3.6	31.6	3.2	1.01	0.02	-068	2.12	0.20
Attention	2.1	0.46	2.0	0.46	0.78	0.00	-0.11	0.25	0.16
Executive function	2.5	1.96	1.3	1.01	3.15	0.43	0.41	1.83	0.75
Memory	3.8	2.61	2.1	1.41	3.47	0.00	0.74	2.73	0.70
Social cognition	1.3	1.46	1.4	1.41	-0.31	0.69	-0.39	-0.69	-0.80
Processing speed	0.74	0.74	0.81	0.78	-0.47	0.63	-0.37	0.61	-0.09

**Figure 2:**
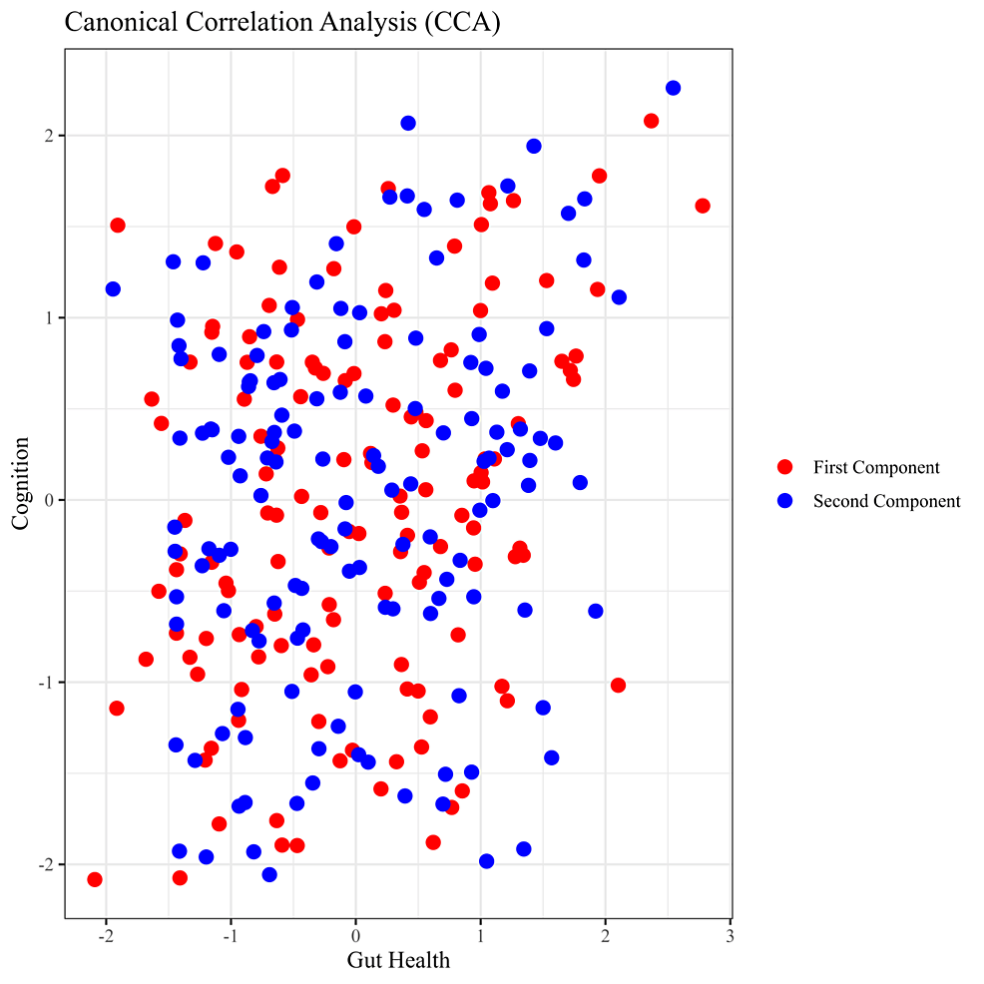
Canonical correlation analysis (CCA) of gut health and cognition

Figure 2 presents the results of a canonical correlation analysis (CCA) examining the relationship between gut health and cognition. The scatter plot displays the first two canonical components derived from the analysis. Each point represents an observation, with red dots corresponding to the first canonical component and blue dots corresponding to the second canonical component. The x-axis represents the gut health scores, while the y-axis represents the cognition scores. The plot illustrates the distribution of the two sets of variables, showing how they correlate within the canonical variables. The spread and clustering of the points suggest the nature of the relationship between gut health and cognitive function, highlighting areas of strong correlation and variability. The CCA plot reveals the relationship between gut health and cognition by projecting the data onto the first two canonical components. The distribution of red and blue points indicates the degree of correlation between the variables. The red points show how the first set of canonical variates, combining gut health and cognition measures, correlate. A more concentrated cluster of red points suggests a stronger linear relationship within this component. The blue points represent the second canonical component, which accounts for additional variability not captured by the first component. The spread of blue points indicates the extent to which other dimensions of the data contribute to the overall relationship. The scatter plot shows that there is a moderate to strong correlation between gut health and cognition, as evidenced by the clustering of points along a diagonal trend. The overlap and spread of the red and blue points suggest that, while there is a significant relationship, other factors and dimensions also play a role in this interaction. This indicates that both gut health and cognitive function are complex, multi-faceted constructs influenced by various underlying factors.

The Appendix (Table 5) also presents the influence of gut health on various aspects of cognitive functioning, including cognition, attention, memory, executive function, processing speed, and social cognition. For overall cognition, gut health accounted for 17% of the variance, but the relationship was not statistically significant (F = 2.3, p = 0.12). This suggests that, while gut health might have some influence on cognitive functioning, the evidence is not strong enough to confirm this effect. In the case of attention, gut health explained only 2% of the variance, with no significant findings (F = 3.1, p = 0.08). This indicates a minimal and non-significant impact of gut health on attention. Memory showed a small but significant influence on gut health, accounting for 1% of the variance with a significant effect (F = 1.3, p = 0.02). This finding suggests a slight but statistically significant relationship between gut health and memory. For executive function, the influence of gut health was negligible, explaining 0% of the variance, with no significant effect (F = 0.20, p = 0.65). This indicates that gut health does not have a measurable impact on executive function. Processing speed showed a more substantial relationship, with gut health accounting for 3% of the variance and a significant effect (F = 4.8, p = 0.03). This suggests that gut health has a significant impact on processing speed. Lastly, for social cognition, gut health explained 1% of the variance, but the relationship was not significant (F = 2.1, p = 0.14). This indicates a minimal and non-significant impact of gut health on social cognition. Overall, the results show that, while gut health has some influence on specific cognitive functions such as memory and processing speed, the effects are generally small and not always statistically significant. The findings highlight the complexity of the relationship between gut health and cognitive functioning, suggesting that other factors may also play a significant role.

## Discussion

Our study aimed to explore the intricate relationship between gut health and cognitive functioning in adults, providing a comprehensive analysis of how various factors associated with gut health influence cognitive performance. The findings from our study add to the growing body of literature on the gut-brain axis, highlighting significant associations between gut health and cognitive functions, particularly memory and processing speed. This discussion delves deeper into these findings, contextualizing them within existing literature and outlining potential implications for future research and practical applications.

According to our research, there is a moderate relationship between gut health and cognitive performance, with substantial relationships seen in the areas of memory and processing speed. This is consistent with earlier studies, such as those by Tooley [22], who discovered links between improved executive function cognitive flexibility and microbial diversity. According to Tooley's [22] narrative review, there may be a way to increase cognition through gut microbiota, as evidenced by research showing improvements in verbal learning and memory, visuospatial memory, and attentional vigilance [22]. Our results underline the need for more research into gut bacteria profiles that support good cognitive health and support the idea that the makeup of the gut microbiota might have a substantial impact on cognitive function. Our results are corroborated by a study by Fekete et al. [23], which shows that prebiotics, probiotics, and symbiotics can enhance several cognitive processes, such as memory, attention, and perception. Their thorough analysis emphasized the gut-brain axis's reciprocal connection and its function in preserving homeostasis [23]. The potential impact of gut microbiota on cognitive health is further supported by the study's finding of strong connections between certain cognitive processes and gut health. These results imply that diet- or supplement-based therapies targeted at modifying gut microbiota may be advantageous for cognitive function.

This study also found significant gender and marital status differences in gut health and cognitive functions. Males exhibited better gut health scores than females, and singles showed better cognitive performance compared to married individuals. These demographic differences highlight the need for considering individual differences when examining the gut-brain axis. For instance, the study by Kong et al. [24] discussed the bidirectional influence of gut microbiota and exercise on brain health, suggesting that lifestyle factors such as physical activity could modulate these relationships. Understanding these individual differences is crucial for developing personalized interventions aimed at improving gut health and cognitive functioning [24]. Moreover, the prospective investigation found that the abundance of a gut bacterial community was positively associated with fluid intelligence scores among healthy young adults [25]. This aligns with our observation that gut health impacts cognitive functions such as memory and processing speed. The positive associations observed in their study suggest that certain gut microbiota compositions can enhance cognitive performance. Our findings extend this knowledge by demonstrating similar associations in a broader adult population, emphasizing the importance of maintaining a healthy gut microbiota for cognitive health.

The systematic review by Kossowska et al. [26] provided insights into the interplay between gut microbiota and cognitive functioning in healthy aging populations. Their findings suggested that changes in gut microbiota composition could serve as early biomarkers for cognitive decline, even in the absence of diagnosed cognitive impairment [26]. This aligns with our observation that gut health impacts cognitive functions, emphasizing the importance of early detection and intervention. By identifying specific gut microbiota profiles associated with cognitive health, we can develop targeted interventions to prevent cognitive decline and promote healthy aging. Our study, like others reviewed in the literature, underscores the potential for gut microbiota-targeted interventions to improve cognitive health. The randomized placebo-controlled trial by Tran et al. [27] demonstrated that probiotics can significantly reduce anxiety and improve mood regulation, suggesting that targeted interventions to modulate gut microbiota could have broad implications for cognitive and mental health [27]. These findings highlight the potential for probiotics to be used as a therapeutic approach for cognitive health, particularly in populations experiencing high levels of stress and anxiety.

The review by Koblinsky et al. [28] emphasized the need for whole diet and exercise interventions that consider microbiota changes and their cognitive effects. Their review highlighted the importance of understanding the underlying mechanisms by which gut microbiota influence cognitive health [28]. Our study's findings contribute to this understanding by demonstrating significant associations between gut health and cognitive functions. The role of the gut microbiome as a component of the gut-brain axis in cognitive health. It also discussed how gut microbiota components and interactions can affect cognitive development and function across the lifespan [29]. Our study's findings align with their review, highlighting the significant influence of gut health on cognitive functions. Understanding these interactions can inform the development of interventions aimed at promoting cognitive health through gut microbiota modulation. Additionally, the cross-sectional study by Meyer et al. [30] found that microbial community composition was associated with cognitive measures in a large community sample. This study emphasized the importance of considering gut microbiota as a factor in cognitive aging [30]. Our findings contribute to this growing body of literature by demonstrating similar associations in an adult population, reinforcing the importance of gut health for cognitive functioning.

This study's strengths include its comprehensive analysis of the gut-brain axis, the use of validated assessment tools, and the identification of significant associations between gut health and cognitive functions, particularly memory and processing speed. The findings suggest that gut microbiota-targeted dietary and lifestyle interventions could enhance cognitive health. Gender and marital status differences underscore the need for personalized approaches in gut-brain research. Future research should aim to replicate these findings in larger, more diverse samples and explore the mechanisms underlying these associations. Investigating gut microbiota-targeted therapies holds promise for developing novel strategies to improve cognitive functioning and overall well-being.

Despite the significant findings of this study, several limitations must be acknowledged. Firstly, the sample size was relatively small, comprising only 140 participants, which may limit the generalizability of the results to the broader population. The study targeted the general population in Islamabad, which may not capture the diversity of gut health and cognitive functioning across different regions and demographics. Secondly, the study relied on self-report questionnaires for both gut health and cognitive assessment, which can introduce biases such as social desirability bias and recall bias. Participants may not accurately remember or report their dietary habits, gastrointestinal symptoms, or cognitive difficulties, potentially affecting the reliability of the data. Additionally, the lack of laboratory tests to directly measure gut microbiota composition is a significant limitation.

## Conclusions

Our research demonstrates the strong correlations that exist between gut health and adult cognitive abilities, including memory and processing speed. These results highlight the possible influence of gut bacteria on cognitive function and are consistent with previous research on the gut-brain axis. The study also stresses how critical it is to take individual differences such as gender and marital status into account when analyzing these links. Overall, the study demonstrated a moderate relationship between gut health and cognitive performance, underscoring the potential impact of gut microbiota on cognitive functioning. According to our findings, specific interventions that modify gut microbiota through food or supplements may be advantageous for cognitive health. This opens exciting new directions for further study and real-world applications in enhancing general cognitive health. Future studies should focus on investigating the causal effects and long-term impact of gut-inducing substances on cognitive health, incorporating controlled trials and longitudinal designs to clarify these intricate associations. Additionally, targeted interventions such as probiotics and dietary modifications hold promise for enhancing cognitive health and preventing cognitive decline. By continuing to explore the gut-brain axis, we can develop more effective strategies for promoting cognitive health and well-being. Future research should also aim to replicate these findings in larger samples and explore the potential for gut microbiota-targeted interventions to improve cognitive health and incorporate laboratory tests alongside self-report measures to enhance the validity and reliability of the findings.

## Appendices

**Table 5:** Influence of gut health on cognitive functioning R=Correlation, R²=Determination, B=Unstandardized Coefficient, β=Standardized Coefficient, SE=Standard Error; p-values calculated using regression analysis; the significance level is set at p < 0.05.

Variable	R	R^2^	B	β	SE	F	p
Cognition	0.13	0.17	14.9	-	4.0	2.3	0.12
Gut health	-	-	-0.19	-1.9	-0.12	-	-
Attention	0.14	0.02	1.5	-	0.35	3.1	0.08
Gut health	-	-	0.01	0.14	0.01	-	-
Memory	0.09	0.01	5.7	-	1.9	1.3	0.02
Gut health	-	-	-0.07	-0.98	0.6	-	-
Executive function	0.03	0.00	2.8	-	1.4	0.20	0.65
Gut health	-	-	-0.02	-0.03	0.04	-	-
Processing speed	1.8	0.03	2.0	-	0.57	4.8	0.03
Gut health	-	-	-0.03	-0.18	0.01	-	-
Social cognition	0.12	0.01	3.0	-	1.1	2.1	0.14
Gut health	-	-	-0.05	-0.12	0.03	-	-

**Table 6:** Gut Health Questionnaire The scale was developed by Muddsar Hameed.

Question	Options	Score
How many meals do you typically consume in a day?	4-5 times a day, 2-3 times a day, 1 time a day	3, 2, 1
How often do you consume fruits and vegetables high in fiber (e.g., apples, broccoli, spinach)?	Daily, Several times a week, Rarely	3, 2, 1
How often do you include whole grains in your diet (e.g., oats, brown rice, quinoa)?	Daily, Occasionally, Rarely	3, 2, 1
How often do you consume foods rich in prebiotics (e.g., onions, garlic, legumes)?	Daily, Occasionally, Rarely	3, 2, 1
Do you include nuts and seeds in your diet (e.g., almonds, chia seeds, flaxseeds)?	Yes, daily, Yes, occasionally, No	3, 2, 1
Do you regularly consume fermented foods (e.g., yogurt, sauerkraut, kefir)?	Yes, daily, Yes, occasionally, No	3, 2, 1
How often do you consume foods high in added sugars and processed fats?	Rarely or never, Occasionally, Frequently	3, 2, 1
How would you rate your overall water intake?	Optimal (8 glasses or more per day), Moderate (4-7 glasses per day), Insufficient (<4/day)	3, 2, 1
Are you currently following any specific dietary restrictions or preferences?	Yes, No	2, 1
How often do you consume probiotic supplements or probiotic-rich foods?	Daily, Occasionally, Never	3, 2, 1
Do you smoke or consume alcohol? If so, how frequently?	Never, Occasionally, Frequently	3, 2, 1
Do you experience any gastrointestinal symptoms (e.g., bloating, gas, constipation, Diarrhea)?	Never, Sometimes, Frequently	3, 2, 1
Do you have any diagnosed gastrointestinal conditions (e.g., IBS, GERD)?	No, Yes	2, 1
How often do you engage in regular physical activity or exercise?	Daily, Several times a week, Rarely or never	3, 2, 1
On a scale of 1 to 10, how would you rate your overall gut health?	10 (Best), 9, 8, 7, 6, 5, 4, 3, 2, 1 (Worst)	10 to 1

**Table 7:** Cognition self-assessment rating scale (C-SARS)

	Rarely	Sometimes	Often	All the time
I have difficulty concentrating on something I am reading, or a TV show I am watching	-	-	-	-
Frequency	-	-	-	-
Impact it has had on your life	-	-	-	-
I have difficulty recalling certain events or what other people had told me	-	-	-	-
Frequency	-	-	-	-
Impact it has had on your life	-	-	-	-
I forget the tasks that I’m supposed to do	-	-	-	-
Frequency	-	-	-	-
Impact it has had on your life	-	-	-	-
I fail to remember where I leave things	-	-	-	-
Frequency	-	-	-	-
Impact it has had on your life	-	-	-	-
Whilst conversating I forget what I was going to speak	-	-	-	-
Frequency	-	-	-	-
Impact it has had on your life	-	-	-	-
I have trouble mapping out a plan for future tasks	-	-	-	-
Frequency	-	-	-	-
How much it interferes with my life	-	-	-	-
Impact it has had on your life	-	-	-	-
Frequency	-	-	-	-
How much it interferes with my life	-	-	-	-
I do tasks without thinking them through first	-	-	-	-
Frequency	-	-	-	-
Impact it has had on your life	-	-	-	-
I find it hard to adapt to change	-	-	-	-
Frequency	-	-	-	-
Impact it has had on your life	-	-	-	-
I lag in mental and physical tasks	-	-	-	-
Frequency	-	-	-	-
Impact it has had on your life	-	-	-	-
I have difficulty deciphering other people's body language	-	-	-	-
Frequency	-	-	-	-
Impact it has had on your life	-	-	-	-
I have trouble gauging other people's feelings and views	-	-	-	-
Frequency	-	-	-	-
Impact it has had on your life	-	-	-	-
